# Buffalo State Insane Asylum

**Published:** 1881-08

**Authors:** 


### Buffalo State Insane Asylum.

### RESIGNATION OF PROF. JAMES P. WHITE AS PRESIDENT OF THE BOARD OF MANAGERS.

At the annual meeting of the Board the following communication was read:

*To the Board of Managers of the Buffalo State Asylum for the Insane:*

More than twelve years since the first petition addressed to the Legislature, asking for the appointment of a commission to locate an Asylum for the Insane in the Eighth Judicial District, was printed and circulated among the medical practitioners in Western New York by myself. This petition was upon application of the physicians in this part of the State numerously signed, and on presentation to the Legislature, then in session, favorably acted upon, and five commissioners appointed by Governor Hoffman to recommend a suitable location for the institution. Five medical men were appointed—Drs. Gray, Baker, Wm. B. Gould, Strong and White—who reported in favor of selecting Buffalo after a sharp competition, in which very liberal offers were made on the part of Lockport, Westfield, Warsaw and Batavia.

After hearing the arguments presented by the advocates of the different places, the Legislature decided to adopt the report of the commissioners recommending Buffalo

The late Mr Joseph Warren had by this time joined me in the undertaking, and upon our representation a bill was passed appropriating fifty thousand dollars to commence the erection of a building, and ten managers were appointed for its care and construction

In May, 1870, the gentlemen appointed organized, electing me to the presidency, to which position your partiality has annually unanimously re-elected me.

During this entire period the success of this great humanitarian institution has been with me paramount in importance to all other considerations. To advance its interests I have at my own expense made ten or twelve journeys to represent to the legislative committees its importance and requirements.

Although the plan of the building may be unnecessarily elaborate and expensive, it is pleasant to believe that for perfection in arrangement, for the classification of patients, for ventilation, and in all the essential requisites of a hospital for the insane it has no superior in any country.

With many delays and embarrassments from various sources, the structure has been in part completed, and is now occupied by the suffering insane The competent and excellent Superintendent and the able corps of assistants, nominated by myself, are successfully carrying forward the good work.

Satisfied that my individual efforts are no longer required to accomplish success, I beg to tender my resignation of the presidency of the Board.

In retiring from the Board and its presidency, I cannot do less than thank you for the many courtesies extended to me during the long period we have been thus associated.

Together we have achieved success and given to Buffalo a most imposing structure for the most benevolent of all purposes—the amelioration of the condition of the helpless insane.

Although admonished by my multifarious duties and occupations that I must diminish my cares, I yet retire from your association with sincere regret, and from a conviction of duty to myself, to other duties and my private engagements.

Respectfully, JAMES P. WHITE.

On motion a committee was appointed by the Chairman to express the sense of the Board in relation to the resignation of Dr. White. They submitted the following:

*Resolved*, That in accepting the resignation of Dr. James P. White as president of the Board of Managers of the Buffalo State Asylum for the Insane, we desire to place on record our sincere regret for the necessity that prompts the step on his part.

*Resolved*, That in thus severing his relations with this Board as the president, he will bear with him our continued confidence and respect for the ability, cheerfulness and devotion with which he has during the past twelve years performed the duties of the office

The resolutions were unanimously adopted.

Francis H. Root was unanimously elected to the position of president, but at first declined to accept. He, however, finally consented to accept for the remainder of the term.

The JOURNAL has ever taken the greatest interest in the progress and success of the Buffalo State Asylum, and we can only join with the managers in expressing "our sincere regret for the necessity that prompts this resignation." It will be received with unfeigned regret by any friend of the institution. More than twelve years ago Dr. White recognized the necessity of an asylum at this end of the State for the unfortunate insane, and the benefits which would accrue to this city by its establishment here. From that day to this he has labored unceasingly with rare tact, ability and judgment to accomplish this purpose. From the first organization of the Board he has been its president, and to-day he points with pride to an asylum of which he can say "for perfection of arrangement, for the classification of patients, for ventilation, and in all the essential requirements of a hospital for the insane, it has no superior in any country." It has now reached a stage when its success is assured, and urged by his friends of the duty which he owes to himself, of to some degree curtailing his labors, and perhaps more strongly by an indisposition, which, we sincerely trust, a much-needed rest from professional and other labors will entirely relieve, "Dr. White retires from the active direction of Asylum affairs." In the words of one of our great city dailies, "If it be true that the culture and christianity of a community are best gauged by the character of the humane institutions, then this city as well as the State of New York owes something to Dr. White, the father and for eleven years president of the Buffalo State Asylum for the Insane."

